# Constitutional *CHEK2* mutations are infrequent in early-onset and familial breast/ovarian cancer patients from Pakistan

**DOI:** 10.1186/1471-2407-13-312

**Published:** 2013-06-27

**Authors:** Muhammad U Rashid, Noor Muhammad, Saima Faisal, Asim Amin, Ute Hamann

**Affiliations:** 1 Shaukat Khanum Memorial Cancer Hospital and Research Centre (SKMCH & RC), Lahore, Pakistan; 2 Deutsches Krebsforschungszentrum (DKFZ), Molecular Genetics of Breast Cancer, Heidelberg, Germany; 3 Levine Cancer Institute, Carolinas Medical Center, Charlotte, USA

**Keywords:** *CHEK2*, Germ line mutations, Early-onset and familial breast cancer, Pakistan

## Abstract

**Background:**

Less than 20% of Pakistani women with early-onset or familial breast/ovarian cancer harbor germ line mutations in the high-penetrance genes *BRCA1*, *BRCA2* and *TP53*. Thus, mutations in other genes confer genetic susceptibility to breast cancer, of which *CHEK2* is a plausible candidate. *CHEK2* encodes a checkpoint kinase, involved in response to DNA damage.

**Methods:**

In the present study we assessed the prevalence of *CHEK2* germ line mutations in 145 *BRCA1/2*-negative early-onset and familial breast/ovarian cancer patients from Pakistan (Group 1). Mutation analysis of the complete *CHEK2* coding region was performed using denaturing high-performance liquid chromatography analysis, followed by DNA sequencing of variant fragments.

**Results:**

Two potentially deleterious missense mutations, c.275C>G (p.P92R) and c.1216C>T, (p.R406C), were identified (1.4%). The c.275C>G mutation is novel and has not been described in other populations. It was detected in a 30-year-old breast cancer patient with a family history of breast and multiple other cancers. The c.1216C>T mutation was found in a 34-year-old ovarian cancer patient from a family with two breast cancer cases. Both mutations were not detected in 229 recently recruited *BRCA1/2*-negative high risk patients (Group 2).

**Conclusion:**

Our findings suggest that *CHEK2* mutations may not contribute significantly to breast/ovarian cancer risk in Pakistani women.

## Background

DNA damage results in activation of cell-cycle checkpoints that inhibit cell proliferation and initiate DNA repair. Impaired function of such checkpoints can lead to genomic instability and susceptibility to cancer. *CHEK2* (cell cycle checkpoint kinase 2, OMIM #604373) is a G2 checkpoint serine/threonine kinase that acts as a tumor suppressor in the nucleus in response to DNA double-strand breakage [1]. The CHEK2 protein is phosphorylated by ataxia-telangiectasia-mutated (ATM) [2] and subsequently phosphorylates critical cell-cycle proteins including TP53, CDC25C, CDC25A and BRCA1, promoting cell cycle arrest, apoptosis and DNA repair [3-6].

Recent studies have indicated that *CHEK2* functions as a moderately effective cancer susceptibility gene; mutations in *CHEK2* predispose individuals to breast cancer [7-10] as well cancer of other sites [11-13]. Thus far, five deleterious recurrent mutations in *CHEK2* have been identified that confer about twofold elevated risk of breast cancer in unselected female population. These include the truncating mutation *CHEK2* c.1100delC [14,15], the missense mutations p.I157T and p.S428F [7,16], the splice site mutation c.IVS2 +1G>A [7,17] and the large genomic 5,395 bp deletion (del5395) [18]. For carriers of the c.1100delC mutation, even higher risk of up to three to five fold was observed among early-onset and familial breast cancer patients [14,15].

The frequencies of these mutations have been shown to vary widely with geographic distribution and ethnicity [19-32]. The prevalence of c.1100delC ranged from being virtually absent in South-European to 1.5% in populations from North-Europe [33], while it was not detected in breast cancer patients from Asia, including China, Korea, Malaysia, Singapore, Japan, the Philippines, India and Pakistan [20-23,28-32]. The variants c.IVS2 +1G>A and del5395 were identified in Poland [7,18], Belarus [17,34], Russia [35] and Czech Republic [36] with a frequency of 0.7%-1.1% and 0.9%-1.3%, respectively; p.S428F was identified in the Ashkenazi Jewish population with a frequency of 2.9% [16] and p.I157T in Finland with a prevalence of 7.4% [26].

The role of *CHEK2* mutations in breast cancer predisposition has been investigated previously in several studies conducted in Asia. Whereas most studies restricted the analysis to the *CHEK2* c.1100delC mutation [20-23,28-32], the study performed by Liu and colleagues included the entire gene [37]. In this study conducted in China a novel pathogenic missense mutation, p.H371Y, was identified in familial and unselected breast cancer cases and controls with a frequency of 4.2%, 1.8% and 0.7%, respectively. These results suggest that *CHEK2* germ line mutations may contribute to breast cancer susceptibility in the Chinese population and point to the need of more whole mutational screens in other Asian populations.

Given the paucity of data on genetic variability of *CHEK2* in Asian populations coupled with the fact that less than 20% of Pakistani women with early-onset or familial breast/ovarian cancer can be attributed to germ line mutations in *BRCA1*, *BRCA2* and *TP53*[38,39], we assessed the prevalence of *CHEK2* mutations in 145 high-risk *BRCA1/2-*negative breast/ovarian cancer patients from Pakistan (Group 1). Comprehensive mutation analysis of the entire coding region and the exon-intron junctions of the *CHEK2* gene were performed. An additional group of 229 high-risk *BRCA1/2*-negative breast/ovarian cancer patients (Group 2) were screened for the identified *CHEK2* mutations.

## Methods

### Study subjects

The study included index patients from 145 breast and/or ovarian cancer families who have been assessed and confirmed to be negative for *BRCA1/2* germ line mutations and described previously (Group 1) [38]. After identification of mutations from the entire coding region of the *CHEK2* gene in this cohort, an additional cohort of 229 *BRCA1/2*-negative high-risk patients was screened for the two identified mutations (Group 2). The index cases were identified using the following criteria: i) families with one female breast cancer diagnosed ≤30 years of age, ii) families with two first- or second-degree (through a male) female relatives diagnosed with breast cancer; at least one diagnosed ≤50 years of age, iii) families with at least three cases of breast cancer; at least one diagnosed ≤50 years of age, iv) families with one male breast cancer case diagnosed at any age, v) families with at least one female breast cancer and one ovarian cancer at any age, vi) families with one ovarian cancer diagnosed ≤45 years of age and vii) families with at least two ovarian cancers; at least one diagnosed ≤45 years of age. Breast and ovarian cancer in the same patient were counted as two independent cases. The number of index cases by phenotype for Group 1 and Group 2 are shown in Table 1.

**Table 1:** **Description of index cases screened for *CHEK2 *mutations**

**Cancer type of index case**	**Phenotype of families**^**a**^	**Number of families**	**Index cases**		**Mean age of index cases in years (age range)**	**Cases with mutations N (%)**
			**Unilateral BC**	**Bilateral BC**		
**Group I**						
Female cases						
BC	Early onset BC (1 case ≤30 years)	66	66	-	27.2 (22–30)	1 (1.5)
BC	HBC (2 cases, ≥1 diagnosed ≤50 years)	30	17	13	34.2 (22–48)	0 (0)
BC	HBC (≥3 cases, ≥1 diagnosed ≤50 years)	9	5	4	38.1 (24–70)	0 (0)
BC	HBOC (≥2 cases)	2	2	-	41.5 (35–48)	0 (0)
BC & OC^b^	HBOC (≥2 cases)	4	3	1	31.2 (29–34)	0 (0)
OC	HBOC (≥2 cases)	2	-	-	31 (28–34)	1 (50)
OC	Early onset OC (1 case ≤45 years)	19	-	-	33.2 (22–45)	0 (0)
OC	HOC (2 cases, ≥1 diagnosed ≤45 years)	2	-	-	34 (31–37)	0 (0)
Male cases						
BC	Male BC	11	11	-	48 (30–73)	0 (0)
All cases^c^		145	104	18		2 (1.4)
**Group II**						
Female cases						
BC	Early onset BC (1 case ≤30 years)	103	103	-	27.1 (19–30)	0 (0)
BC	HBC (2 cases, ≥1 diagnosed ≤50 years)	55	49	6	40.8 (19–61)	0 (0)
BC	HBC (≥3 cases, ≥1 diagnosed ≤50 years)	53	47	6	44.4 (26–73)	0 (0)
BC	HBOC (≥2 cases)	8	7	1	49 (26–67)	0 (0)
BC & OC^b^	HBOC (≥2 cases)	3	2	1	45.6 (30–59)	0 (0)
OC	HBOC (≥2 cases)	5	-	-	43.2 (33–60)	0 (0)
OC	Early onset OC (1 case ≤45 years)	2	-	-	30.5 (25–36)	0 (0)
All cases^d^		229	208	14		0 (0)

*BC*, breast cancer; *HBC*, hereditary breast cancer; *HBOC*, hereditary breast and ovarian cancer; *HOC*, hereditary ovarian cancer; *OC*, ovarian cancer.

^a^see “Study subjects in Method section”.

^b^Breast and ovarian cancer in the same patient were counted as two independent cases.

^c^Including 111 female BCs, 11 male BCs and 27 OCs.

^d^Including 222 female BCs and 10 OCs.

Control population comprised 150 healthy females of Pakistani origin. These were either attendants of hospital registered patients or visiting the cancer centre for medical reasons other than cancer. All study participants were furnished with and signed an informed written consent. The study was approved by the Institutional Review Board of the SKMCH & RC.

### Mutation screening

Genomic DNA was extracted as previously described [38]. The entire coding region and exon-intron junctions of the *CHEK2* gene (Genbank accession number NM_007194.2) were screened in the 145 index patients using denaturing high-performance liquid chromatography (DHPLC) analysis. DHPLC analysis was carried out with the WAVE system (Transgenomics, Omaha, NE). Since multiple homologous copies are present for exons 10–14 of this gene, a nested PCR approach was employed as previously described [40]. PCR-primer pairs and DHPLC running conditions for exon 1 and exons 4–14 were according to Dufault and colleagues [41] and for exons 2 and 3 according to Kleibl and colleagues [42]. When available, a mutation positive control for each exon was included in the DHPLC analyses.

The presence of the *CHEK2* mutations identified in Group 1 was subsequently assessed in the 229 *BRCA1/2*-negative high-risk cases of Group 2 and in 150 healthy female controls.

### DNA sequence analysis

Each sample showing variants detected by DHPLC analysis was sequenced using an automated DNA CEQ 8000 sequencer (Beckman, Hilden, Germany) according to the manufacturers’ instructions. Bi-directional genomic DNA sequencing was performed to verify the presence of a mutation.

### *In silico* analyses

*CHEK2* missense variants (p.P92R; p.R406C) were analyzed for their potential effect on protein function using the default settings of web tools Align-GVGD (http://agvgd.iarc.fr/agvgd_input.php), PolyPhen2 (http://genetics.bwh.harvard.edu/pph2), SIFT (http://sift.jcvi.org) and SNAP (http://www.rostlab.org/services/snap).

## Results

We studied 145 unrelated affected index patients from Pakistani breast and/or ovarian cancer families all of whom have previously been tested and found to be negative for *BRCA1/2* germ line mutations (Group 1). The median age of onset of disease was 29 years (range 22–70) for female breast cancer (n = 111), 45 years (range 30–73) for male breast cancer (n = 11) and 33 years (range 22–45) for ovarian cancer (n = 27). Eighteen of 111 (16.2%) women were diagnosed with bilateral breast cancer. Five cases reported a relative affected with colorectal or prostate cancer.

The joint approach of DHPLC and DNA sequencing analysis revealed ten *CHEK2* sequence variants: one silent mutation, three missense mutations and six intronic variants (Table 2).

**Table 2:** ***CHEK2 *germ line mutations and nucleotide changes in non- *BRCA1/2 *-associated early-onset and familial breast/ovarian cancer patients from Pakistan**

**Location**	**Coding (c.) DNA sequence**^**a**^	**Effect**	**SNP Link**^**b**^	**Classification**^**c**^	**Prevalence**		
					**Group 1**	**Group 2**	
					**Cases (n = 145) n (%)**	**Cases (n = 229) n (%)**	**Controls (n = 150) n (%)**
Exon 1	c.252A>G (E84E)	Silent	rs1805129	P	13 (8.9)	18 (7.9)	12 (8.0)
**Exon 1**	**c.275C>G (P92R)**	**Missense**	**-**	**M**	**1 (0.7)**	**0 (0)**	**0 (0)**
Exon 10	c.1216C>T (R406C)	Missense	-	M	1 (0.7)	0 (0)	0 (0)
Exon 13	c.1501G>A (E501K)	Missense	rs17883172	P	3 (2.1)	1 (0.4)	0 (0)
Intron 1	c.319 +43_319 +44insA	Intronic	rs17879991	P	64 (44.1)	**-**	47 (31.3)
Intron 3	c.592 +50A>T	Intronic	rs17881298	P	1 (0.7)	**-**	0 (0)
**Intron 4**	**c.683 +119_683 +122delATTT**	**Intronic**	**-**	**P**	**2 (1.4)**	**-**	**1 (0.7)**
Intron 4	c.684 -78_-100dup23	Intronic	-	P	16 (11.0)	**-**	20 (13.3)
**Intron 7**	**c.908 +48dupA**	**Intronic**	**-**	**VUS**	**1 (0.7)**	**-**	**0 (0)**
**Intron 13**	**c.1542 +92dupA**	**Intronic**	**-**	**VUS**	**1 (0.7)**	**-**	**0 (0)**

Novel germ line mutations and nucleotide changes are marked in bold.

*P*, polymorphism; *M*, mutation; *VUS*, variant of unknown significance.

^a^Nomenclature follows Human Genome Variation Society (HGVS) (http://www.hgvs.org). Numbering starts at the first A of the first coding ATG (located in exon 2) of NCBI GenBank Accession NM_007194.

^b^Link to NCBI SNP database (http://ncbi.nlm.nih.gov/projects/SNP/).

^c^Classification of missense mutations is based on *in silico* analyses.

Two missense potentially functional mutations, c.275C>G (p.P92R) and c.1216C>T (p.R406C) were identified in 2 out of the 145 (1.4%) breast/ovarian cancer families (Table 1). c.275C>G (p.P92R) is a novel mutation that has not previously been reported in other populations (Additional file 1: Figure S1). It was detected in a 30-year-old early-onset breast cancer patient (IV:3) of Punjabi ethnicity, who presented with a grade 3, lymph node positive, invasive ductal carcinoma, positive for expression of estrogen receptor (ER), progesterone receptor (PR) and epidermal growth factor receptor 2 (HER2) (data not shown). The mutation was also found in another distant paternal female relative, who was diagnosed with breast cancer at 53 years of age (III:9) (Additional file 2: Figure S2A). On the paternal side of the index patient, two more female breast cancers, one bilateral disease diagnosed at 30 and 60 years of age and the other at unknown age, one intestinal cancer, one lung cancer, one cancer of unknown type and one Hodgkin lymphoma were reported. To predict the significance of the novel mutation *in silico* analyses were performed using the computational tools Align-GVGD, PolyPhen2, SIFT and SNAP. The mutation was predicted to be likely pathogenic by PolyPhen2 and SNAP.

The other missense mutation c.1216C>T (p.R406C) was found in a patient from Punjab, who was diagnosed with ovarian cancer at age 34 (IV:2). The mother of the index patient and a maternal aunt of the mother were diagnosed with breast cancer at 38 years of age and at unknown age, respectively (Additional file 2: Figure S2B). The mutation carrier presented with a high grade papillary serous carcinoma. This germ line mutation has previously been reported in two female breast cancer patients [43,44] and was predicted to be likely pathogenic *in silico*[43].

Both missense mutations are rare as they were not detected in Group 2 that included 229 patients with *BRCA1/2*-negative breast/ovarian cancer or the 150 healthy female controls indicating that they may be disease-causative.

Of the other described *CHEK2* variants, the silent mutation, one missense mutation and three intronic variants have been previously classified as polymorphisms (Table 2). Three of these (c.252A>G, c.319 +43_319 +44insA and c.592 +50A>T) have also been identified in the NHLBI Exome Sequencing Project (http://evs.gs.washington.edu/EVS/). Three novel variants are located in introns and are deemed unlikely to have a functional effect.

## Discussion

The relevance of *CHEK2* mutations as a screening target for an elevated risk of breast cancer is of interest. Given the variability among different populations the frequency within a given population needs to be ascertained to qualify relevance. Our contribution to this area of investigation was to establish relevance in the *BRCA1/2*-negative high-risk breast/ovarian cancer Pakistani population by assessing the prevalence of *CHEK2* mutations. While only one Asian study conducted in China has investigated the frequency of *CHEK2* germ line mutations using the whole gene screen [37], our study will provide additional information on genetic variability of the *CHEK2* gene in an Asian population from Pakistan.

The risk conferred by *CHEK2* germ line mutations to breast and ovarian cancer in Asian populations is not well defined since most studies have determined the frequency of the c.1100delC mutation but did not evaluate other mutations. This holds true for studies conducted in Korea [23], Malaysia [31], Singapore [28], Japan [21], China [22,32], India [29,30], Pakistan and the Philippines [20]. In the present study we investigated the entire *CHEK2* coding sequence including the exon-intron junctions using DHPLC analysis followed by direct DNA sequencing of variant fragments. Two potentially deleterious missense mutations, novel p.P92R and p.R406C, were identified in two unrelated patients. Both mutations are rare as they were not detected in a second group of 229 *BRCA1/2*-negative early-onset and familial breast/ovarian cancer patients and 150 healthy controls suggesting that they may be associated with the disease. Our findings suggest that *CHEK2* missense mutations may not contribute significantly to breast/ovarian cancer susceptibility in Pakistan. In a study conducted in China that looked at *CHEK2* mutations based on whole gene analysis, a novel recurrent missense mutation, p.H371Y, was identified in 5/118 (4.2%) familial and 16/909 (1.8%) unselected breast cancer patients and in 9/1,228 (0.7%) controls [37]. The results of this study and the present one suggest that *CHEK2* germ line mutations may contribute to breast cancer susceptibility in populations from East Asia, but may play a minor role in a South Asian population from Pakistan. Our data on the absence of the c.1100delC mutation in Pakistan are in line with those of previous studies [20-23,28-32] supporting the notion that c.1100delC does not contribute to breast cancer susceptibility in Asian populations.

In contrast to the single study of a whole *CHEK2* gene screen in an Asian population, many studies have been performed in Caucasian populations reporting different mutations and mutation frequencies in the range from 0% to 12%. In the French-Canadian population no mutations were detected in 25 (0%) *BRCA1/2*-negative familial breast cancer patients [19]. In France mutations were found in 15/507 (2.9%) *BRCA1/2-*negative familial breast cancer cases [25]. In the United States mutations were observed in 5/169 (3%) early-onset breast cancer patients (≤40 years) [21] and in another study mutations were identified in 51/1,303 (3.9%) early-onset patients (≤45 years) from the United States, Canada and Australia [43]. The highest mutation frequencies were reported in 30/516 (5.8%) *BRCA1/2*-negative familial breast cancer patients from Germany [41], 10/172 (5.8%) Ashkenazi Jewish familial *BRCA1/2*-founder mutation-negative breast and/or ovarian cancer patients or unaffected women [45], 8/89 (8.9%) familial breast cancer patients from the UK, North America and the Netherlands [46] and in 10/82 (12.2%) familial *BRCA1/2*-founder mutation-negative breast and/or ovarian cancer patients from Finland [27].

Mutation screening was performed using the combined approach of DHPLC analysis followed by DNA sequencing of variant fragments. DHPLC has been commonly used for mutation screening of various genes as it is rapid, cost-effective and highly sensitive detecting 93 to 100% of mutations that were observed by DNA sequencing [47]. However, since the DHPLC mutation detection rate is not 100% for each gene, it cannot be ruled out that some mutations were missed. *CHEK2* mutation analysis is hampered by the presence of multiple homologous copies for exons 10–14 and requires an adapted primer design and amplification conditions. In the present study we employed a nested PCR strategy that specifically amplified the functional copy of the *CHEK2* gene [40].

The *CHEK2* mutation frequency of 2/145 (1.4%) observed in this study may be an underestimate as the sensitivity of DHPLC can be below 100% and screening for regulatory mutations residing outside the coding region and for large genomic rearrangements was not performed. Previously a large genomic deletion of 5,395 bp, which results in loss of exons 9 and 10, has been identified in two families of Czechoslovakian ancestry, in a series of Czech and Slovak breast cancer patients (8/631, 1.3%) enriched for familial cases and was absent in 367 controls [36]. In Poland the same deletion was detected in 19/1,978 (1.0%) unselected breast cancer cases, 28/3,228 (0.9%) early-onset cases and in 24/5,496 (0.4%) controls [18]. Thus further analyses for genomic rearrangements in Asian populations may reveal additional *CHEK2* mutations as well.

In the present study a novel missense mutation, p.P92R, was identified in a young Pakistani breast cancer patient of Punjabi origin from a family with multiple breast cancers, intestinal cancer, lung cancer, cancer of unknown type and Hodgkin lymphoma. The mutation was also found in another paternal female relative diagnosed with breast cancer at 53 years of age. Since *CHEK2* is known to be a multi-organ cancer susceptibility gene [7], the mutation may have co-segregated with the disease in this family. However, this could not be tested since DNA samples of relevant family members were not available for analysis. The other mutation, p.R406C, was found in a young Punjabi patient diagnosed with ovarian papillary serous carcinoma, who reported a family history of breast cancer. The same mutation has previously been observed in a Spanish familial breast cancer patient and in an early-onset breast cancer patient (≤45 years) of unknown ancestry, but was absent in 400 healthy controls from Spain and 1,109 controls from Canada, the USA and Australia [43,44]. Due to the lack of DNA samples of relevant family members, co-segregation of this mutation with cancer could not be studied.

The breast tumor of the patient harboring the p.P92R missense mutation was positive for ER, which is consistent with previous findings that breast tumors linked with *CHEK2* frame shift and missense mutations (c.1100delC, c.IVS2 +1G>A, del5395, p.I157T) are predominantly ER-positive [48-50]. Additionally, an association of the *CHEK2* p.I157T missense mutation has been reported with lobular carcinoma [50,51]. This link was not observed with the novel p.P92R missense mutation in the present study given the only p.P92R-associated tumor was invasive ductal carcinoma. Given the solitary finding, no interpretation could be rendered.

Our study included cases and controls from only one ethnic group providing uniformity of subjects, adding to the robustness of data. All patients had been selected for *BRCA1/2* testing by virtue of having an early-onset of disease or a family history suggestive of hereditary breast/ovarian cancer [38]. Genetic variability in *CHEK2* was analyzed in a whole gene screen and was not restricted to the few regions with known *CHEK2* mutations. Limitations of the present study are that the functional effects p.P92R and p.R406C and the frequency of large genomic rearrangements were not investigated.

## Conclusions

In this first study of the prevalence of *CHEK2* germ line mutations in 145 *BRCA1/2*-negative early-onset and familial breast and/or ovarian cancer from Pakistan two potentially deleterious mutations were identified. Both mutations are rare as they were not found in 229 other high-risk patients. Our findings suggests that *CHEK2* germ line mutations play a negligible role in early-onset and familial breast/ovarian cancer in Pakistan and imply that *CHEK2* mutation screening of high-risk patients is not warranted in this population.

## Abbreviations

CHEK2: Cell cycle checkpoint kinase 2 gene; DHPLC: Denaturing high-performance liquid chromatography.

## Competing interests

The authors declare that they have no competing interests.

## Authors’ contributions

MUR contributed to conception and design of the study, patient recruitment and data acquisition. In addition, he was involved in data analysis, interpretation and in drafting and revising the manuscript. NM performed the molecular analyses and contributed to data analysis and interpretation. SF was involved in patient recruitment and data acquisition. UH contributed to conception and design of the study, data analysis and interpretation and led the writing of the manuscript. All authors read and approved the final manuscript.

## Pre-publication history

The pre-publication history for this paper can be accessed here:

http://www.biomedcentral.com/1471-2407/13/312/prepub

## Supplementary Material

Additional file 1: Figure S1 DNA mutation analysis of *CHEK2* c.275C>G (P92R). DNA sequencing chromatograms of the forward strand showing the region containing the c.275C>G sequence of healthy control sample (**A**) and corresponding interval from DNA of a c.275C>G mutation carrier (**B**). The arrow indicates the position of the mutation in the chromatogram. S, C>G. Click here for file

Additional file 2: Figure S2 Pedigrees of *CHEK2* c.275C>G (**A**) and c.1216C>T (**B**) mutation carrier Families 112 and 171. *Circles* are females, *squares* are males, and a *diagonal slash* indicates a deceased individual. Symbols with *filled left upper quadrant*: unilateral breast cancer. Symbols with *filled upper half circle*: bilateral breast cancer. Symbols with *filled left lower quadrant*: ovarian cancer. Symbols with *filled right lower quadrant*: cancer other than breast cancer, cancer type is indicated. Identification numbers of individuals are below the symbols. The index patient is indicated by an *arrow*. *A,* age; *BC,* breast cancer; *HD*, Hodgkin’s disease; *OC,* ovarian cancer; *CA*, cancer; *D,* death. The numbers following these abbreviations indicate age at recruitment, age at cancer diagnosis and age at death. *M+,* mutation positive. *M-,* mutation negative. Click here for file
