# Supplementary material for: Constitutional CHEK2 mutations are infrequent in early-onset and familial breast/ovarian cancer patients from Pakistan
Source: BMC Cancer. 2013 Jun 27;13:312. doi: 10.1186/1471-2407-13-312 (PMC3699428; doi:10.1186/1471-2407-13-312)
Supplement: Additional file 1: Figure S1 — DNA mutation analysis of CHEK2 c.275C>G (P92R). DNA sequencing chromatograms of the forward strand showing the region containing the c.275C>G sequence of healthy control sample (A) and corresponding interval from DNA of a c.275C>G mutation carrier (B). The arrow indicates the position of the mutation in the chromatogram. S, C>G. [file 1471-2407-13-312-S1.doc]

**Supplementary Figure 1**

**A**


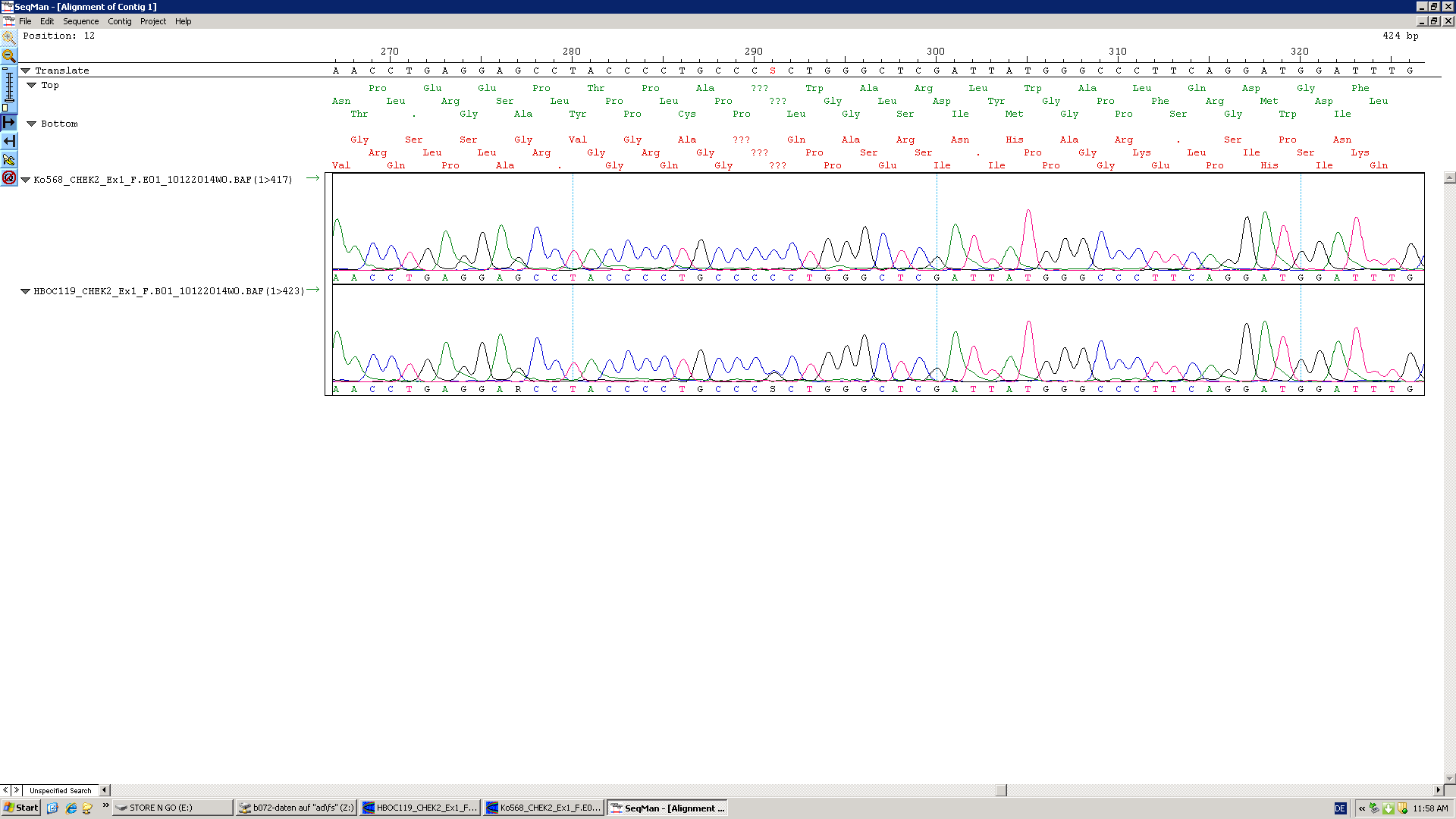


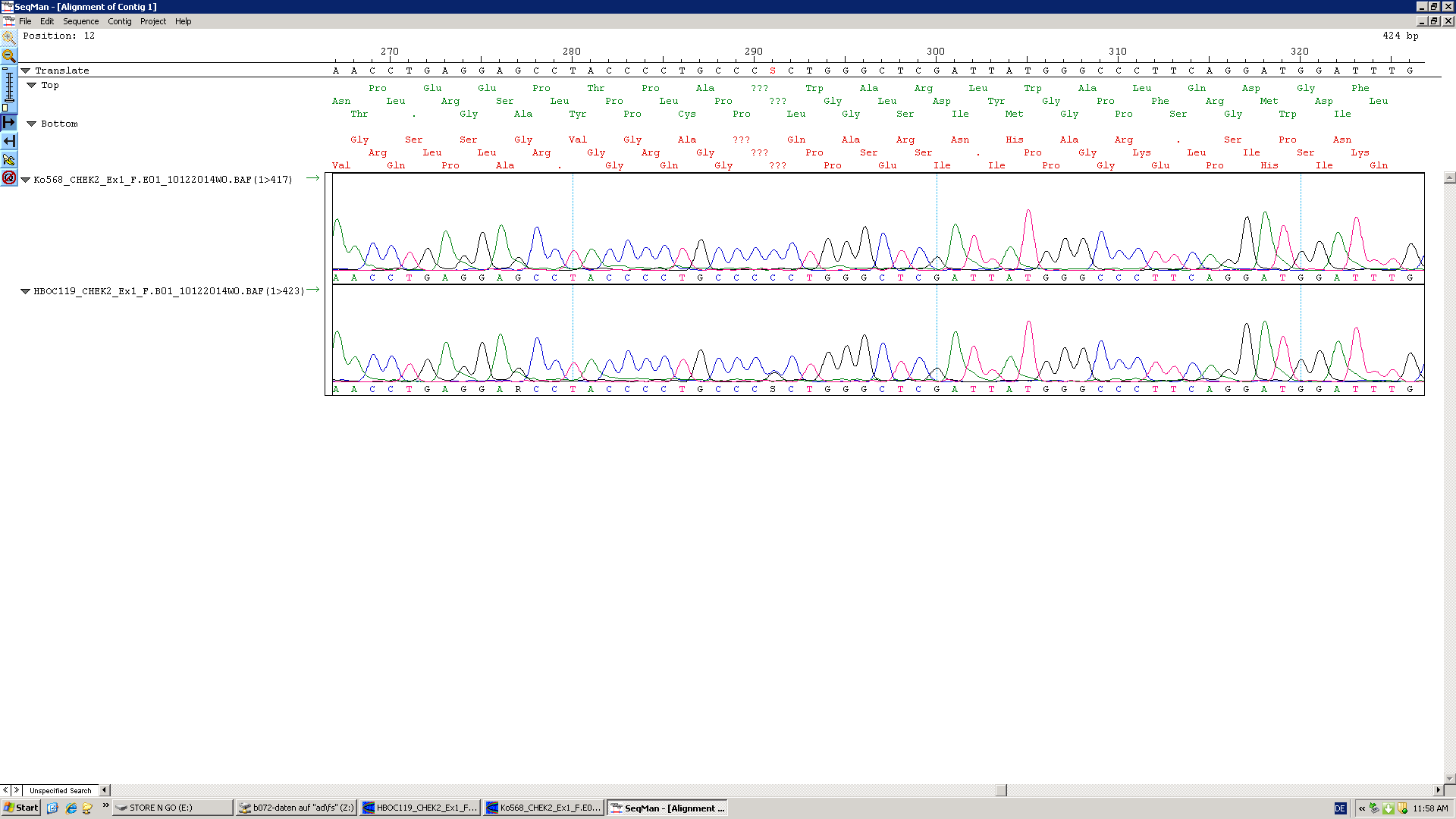


**B**


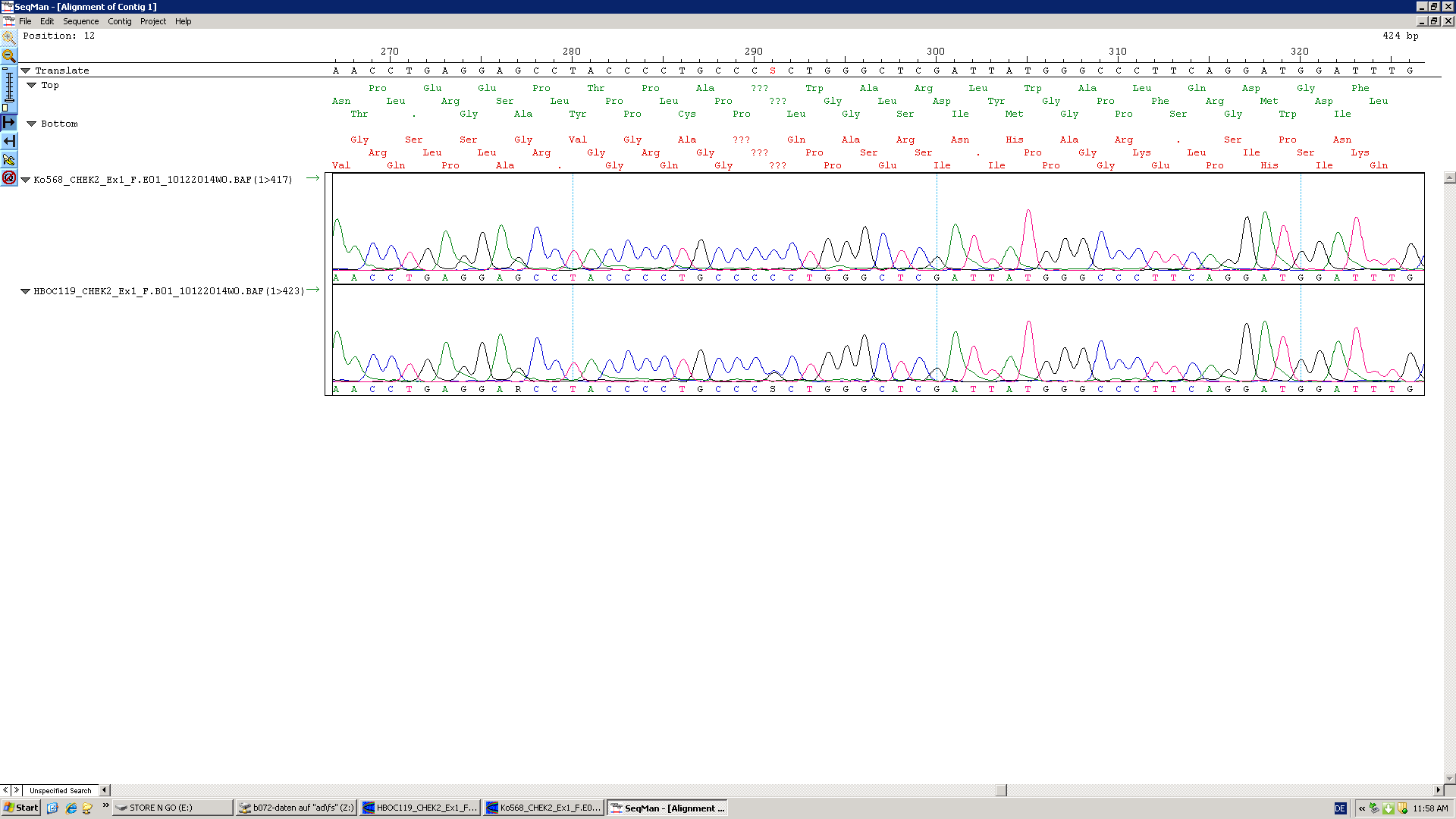


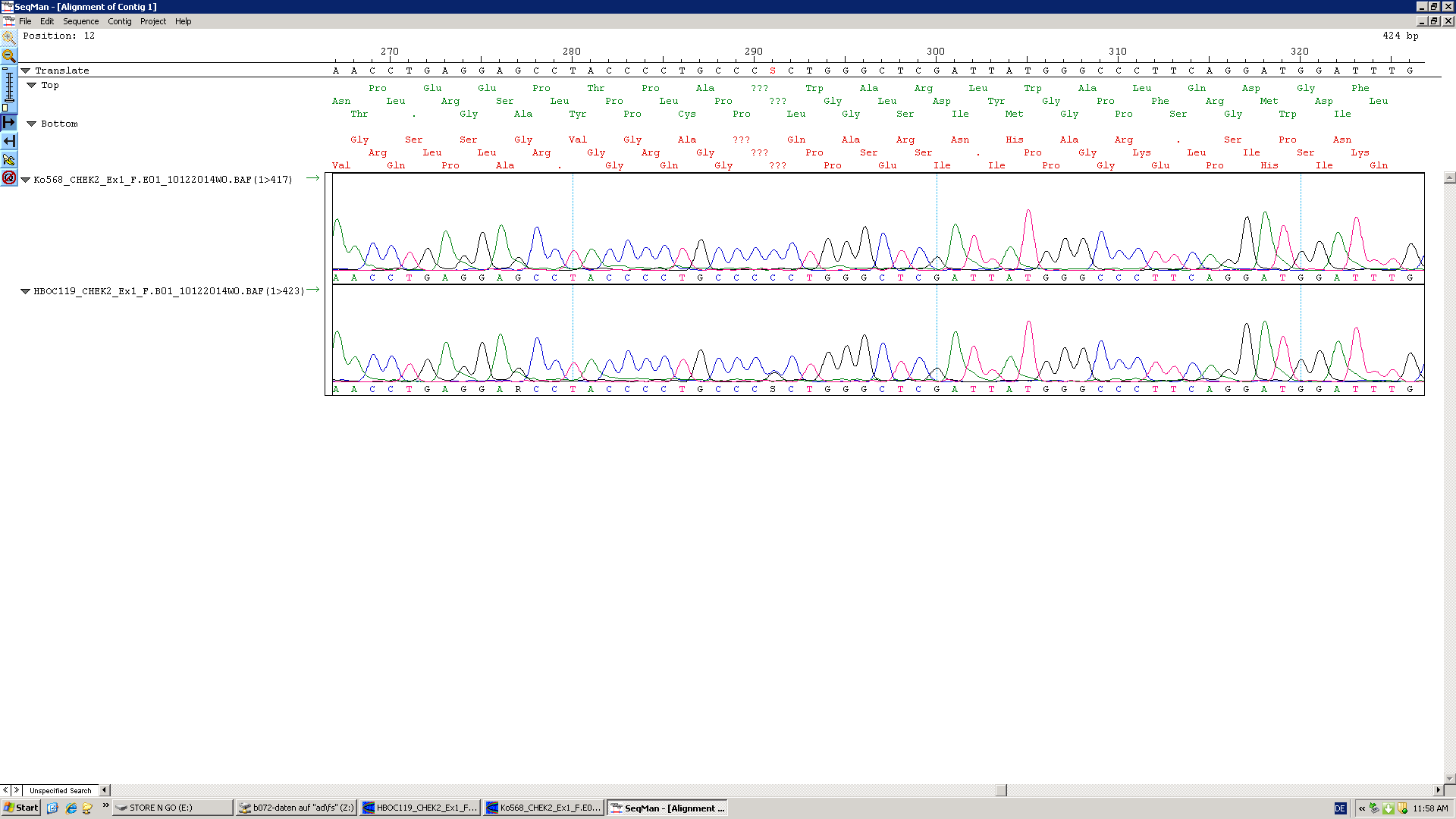


**Supplementary Figure 1 DNA mutation analysis of *CHEK2* c.275C>G (P92R).** DNA sequencing chromatograms of the forward strand showing the region containing the c.275C>G sequence of healthy control sample **(A)** and corresponding interval from DNA of a c.275C>G mutation carrier **(B)**. The arrow indicates the position of the mutation in the chromatogram. S, C>G.
