# Supplementary material for: Constitutional CHEK2 mutations are infrequent in early-onset and familial breast/ovarian cancer patients from Pakistan
Source: BMC Cancer. 2013 Jun 27;13:312. doi: 10.1186/1471-2407-13-312 (PMC3699428; doi:10.1186/1471-2407-13-312)
Supplement: Additional file 2: Figure S2 — Pedigrees of CHEK2 c.275C>G (A) and c.1216C>T (B) mutation carrier Families 112 and 171. Circles are females, squares are males, and a diagonal slash indicates a deceased individual. Symbols with filled left upper quadrant: unilateral breast cancer. Symbols with filled upper half circle: bilateral breast cancer. Symbols with filled left lower quadrant: ovarian cancer. Symbols with filled right lower quadrant: cancer other than breast cancer, cancer type is indicated. Identification numbers of individuals are below the symbols. The index patient is indicated by an arrow. A, age; BC, breast cancer; HD, Hodgkin’s disease; OC, ovarian cancer; CA, cancer; D, death. The numbers following these abbreviations indicate age at recruitment, age at cancer diagnosis and age at death. M+, mutation positive. M-, mutation negative. [file 1471-2407-13-312-S2.doc]

**Supplementary Figure 2**

**A.** Family 112, carrier of *CHEK2* c.275C>G (p.P92R)

**B.** Family 171, carrier of *CHEK2* c.1216C>T (p.R406C)

**Supplementary Figure 2**  **Pedigrees of *CHEK2* c.275C>G (A) and c.1216C>T (B) mutation carrier Families 112 and 171**. *Circles* are females, *squares* are males, and a *diagonal slash* indicates a deceased individual. Symbols with *filled* *left upper quadrant*: unilateral breast cancer. Symbols with *filled upper half circle*: bilateral breast cancer. Symbols with *filled left lower quadrant*: ovarian cancer. Symbols with *filled right lower quadrant*: cancer other than breast cancer, cancer type is indicated. Identification numbers of individuals are below the symbols. The index patient is indicated by an *arrow*. *A,* age; *BC,* breast cancer; *HD*, Hodgkin’s disease; *OC,* ovarian cancer; *CA*, cancer; *D,* death. The numbers following these abbreviations indicate age at recruitment, age at cancer diagnosis and age at death. *M+,* mutation positive. *M-,* mutation negative.
